# A System of Surgery

**Published:** 1896-03

**Authors:** 


A SYSTEM OF SURGERY. By American authors. Edited by FREDERIC S. DENNIS, M. D., Professor of the Principles and Practice of Surgery, Bellevue Hospital Medical College, New York; President of the American Surgical Association, etc.; assisted by John S. Billings, M. D., LL. D., D. C. L., Deputy Surgeon-General, U. S. A. To be completed in four imperial octavo volumes, containing about 900 pages each, with index. Profusely illustrated with figures in colors and in black. Volume III., 908 pages, 207 engravings and ten colored plates. Price, per volume, $6.00 in cloth; $7.00 in leather; $8.50 in half-morocco, gilt back and top. For sale by subscription. Full circular free to any address on application to the publishers, Lea Brothers & Co., Philadelphia.

Volume III. of Dennis's Surgery is devoted largely to the various specialties. In it one finds the diseases of the larynx, eye, ear, skin, genito-urinary organs, syphilis, etc., considered by specialists of international repute in their respective lines.

The articles upon the ear, eye and skin are all concise but complete monographs. The chapter upon the ear by Dr. Gorham Bacon is a most admirable presentation of a subject which is always of great interest to the general surgeon. The peculiar value of this article lies in the fact that it has been written by one who is a general surgeon as well as an ear specialist. We find, therefore, not only the diseases of the ear, but such sequelæ as mastoid diseases, brain abscess and sinus-thrombosis thoroughly treated. In limited compass the author has given us an article along the lines laid down by Macewen in his recent masterly work upon the diseases of the brain and spinal cord. We cannot refrain from expressing the wish that there might be more specialists like the author whose writings deserve a place in the library of every general surgeon.

Another most instructive and able chapter is that upon genito-urinary diseases, by Dr. White. In the pages of this article may be found a large fund of knowledge and experience, combined with a terse and happy method of expression, for which the author is justly noted. The whole tone of the chapter is positive and convincing, and although frequently radical will be found safe and useful in its teaching. The article on syphilis by Dr. Taylor is a most admirable exposition of this interesting and important subject. All the salient features of the malady have been concisely and judiciously combined and presented so as to give the writer a pretty thorough knowledge of the subject. The volume is, of course, similar in dress to those preceding, and in every way maintains the enviable reputation of the world-known house which has issued it. J. P.

